# Altering Cell-Cell Interaction in Prenatal Alcohol Exposure Models: Insight on Cell-Adhesion Molecules During Brain Development

**DOI:** 10.3389/fnmol.2021.753537

**Published:** 2021-12-15

**Authors:** Valentina Licheri, Jonathan L. Brigman

**Affiliations:** ^1^Department of Neurosciences, University of New Mexico School of Medicine, Albuquerque, NM, United States; ^2^New Mexico Alcohol Research Center, UNM Health Sciences Center, Albuquerque, NM, United States

**Keywords:** prenatal alcohol exposure (PAE), fetal alcohol spectrum disorder (FASD), cell adhesion molecule (CAM), central nervous system development, cell interaction

## Abstract

Alcohol exposure during pregnancy disrupts the development of the brain and produces long lasting behavioral and cognitive impairments collectively known as Fetal Alcohol Spectrum Disorders (FASDs). FASDs are characterized by alterations in learning, working memory, social behavior and executive function. A large body of literature using preclinical prenatal alcohol exposure models reports alcohol-induced changes in architecture and activity in specific brain regions affecting cognition. While multiple putative mechanisms of alcohol’s long-lasting effects on morphology and behavior have been investigated, an area that has received less attention is the effect of alcohol on cell adhesion molecules (CAMs). The embryo/fetal development represents a crucial period for Central Nervous System (CNS) development during which the cell-cell interaction plays an important role. CAMs play a critical role in neuronal migration and differentiation, synaptic organization and function which may be disrupted by alcohol. In this review, we summarize the physiological structure and role of CAMs involved in brain development, review the current literature on prenatal alcohol exposure effects on CAM function in different experimental models and pinpoint areas needed for future study to better understand how CAMs may mediate the morphological, sensory and behavioral outcomes in FASDs.

## Introduction

Maternal alcohol consumption during pregnancy is well recognized as an important public health concern. In the United States, 1 in 9 pregnant women drink alcohol (May et al., 2009; Tan et al., 2015; Fontaine et al., 2016; Popova et al., 2017), while almost 16% of European women consume alcohol during pregnancy (Mårdby et al., 2017). Approximately 20 years ago, the term Fetal Alcohol Spectrum Disorders (FASDs) was introduced to recognize the broad range of effects induced by maternal alcohol exposure (Koren et al., 2003; Sokol et al., 2003; Chudley et al., 2005; Cook et al., 2016). FASDs are characterized by impairments in working memory, response inhibition, and behavioral flexibility (Streissguth et al., 1991; Mattson et al., 1999; Green et al., 2009; Marquardt et al., 2020).

### Fetal Alcohol Spectrum Disorder Symptoms

The symptoms of FASDs are classified into three categories including craniofacial malformations, sensory and cognitive abnormalities, and brain structure anomalies. Both clinical and preclinical studies have demonstrated that prenatal alcohol exposure (PAE) can induce craniofacial anomalies including a flat nasal bridge, an upturned and short nose, thin upper lip, a smooth philtrum, and micrognathia (Moore et al., 2002, 2007; Wattendorf and Muenke, 2005). Moreover, FASD infants commonly exhibit low body weight, short height and smaller head size (Murawski et al., 2015).

Abnormalities in sensory processing including taste, smell and tactile sensitivity are reported after PAE (Franklin et al., 2008; Bower et al., 2013). In addition, vision and auditory processes can be affected, including symptoms such as microphthalmia with reduced palpebral fissure length, convergent strabismus and low visual acuity (Strömland and Pinazo-Duran, 2002; Strömland et al., 2015) and hearing loss (Tesche et al., 2014; Yoshida et al., 2018). While both changes in morphology and sensory systems are commonly seen when level of exposure is high, if has become increasingly clear that impairments in executive functioning, memory and attention can be present even without the hallmark physical changes seen in Fetal Alcohol Syndrome (FAS) (Mattson et al., 1997, 2011; Bertrand et al., 2005).

In the last decade, studies have reported fine and gross motor deficits, poorer manual coordination and balance problems in children with FASD (Doney et al., 2014; Taggart et al., 2017). Moreover, neuroimaging studies have revealed several changes in brain structure including hypoplasia of the corpus callosum and cerebellum (Chen et al., 2012; Colby et al., 2012; Yang et al., 2012; Boronat et al., 2017), and reduction in overall cortical volume in those with FASD (Rajaprakash et al., 2014).

In addition to commonly reported alterations in brain morphology in PAE, studies have also described correlations between alcohol consumption during pregnancy and anomalies in other organs including heart, kidney, liver, and endocrine system (Caputo et al., 2016). Congenital heart defects, structural anomalies of the heart and great vessels have also been observed in children with FASD (Yang et al., 2015). The correlation between PAE, kidney, and liver is still not well understood, as studies performed have generally reported non-specific anomalies with FASD such as kidney hypoplasia and hydronephrosis and liver hyperbilirubinemia (Hofer and Burd, 2009).

### Alcohol Consumption During Pregnancy

The global prevalence of FASDs is ∼1% of the general population (Popova et al., 2016; Lange et al., 2017), with rates in the US of ∼2–5% (May et al., 2009, 2013, 2014, 2015), and an estimated prevalence in Europe of ∼2% (Lange et al., 2017). The highest numbers of FASD cases reflect the high alcohol consumption, in fact the 7.3% of pregnancies are alcohol-exposed (Green et al., 2016), considering that the 45% of pregnancies are unplanned and/or unrecognized during the first days (Finer and Zolna, 2016; Wozniak et al., 2019).

The amount, pattern and the timing of alcohol consumption during the pregnancy are critical factors in determining the impact of PAE on development. Data from animal models demonstrate a clear correlation between drinking pattern and the effects of PAE on brain size and volume. Bonthius and West conducted a study comparing different drinking patterns in which three groups of newborn rats were exposed to alcohol during the early postnatal period (PND 4–10). The first group was exposed to 4.5 g/kg/day for 4 h per day, while the second received the same dose, but for 8 h per day. The last group received a higher dose of alcohol (6.6 g/kg/day) for 24 h. Interestingly, the results showed that a lower daily dose was more dangerous than a higher consumed in 24 h, as brain weights from the first experimental group were significantly lower than the third group (Bonthius and West, 1990). Human studies support these conclusions as maternal binge drinking during the month prior to pregnancy recognition has been found to correlate with neurobehavioral deficits in attention, memory and in cognitive flexibility in children at age 7.5 (Streissguth et al., 1990).

Recent neuroimaging studies have also shown that children and adolescents exposed to heavy alcohol consumption *in utero* (more than seven drinks/week) have anomalies in cortical thickness and reductions in brain volume (Donald et al., 2015; Robertson et al., 2016; Hendrickson et al., 2018; Treit and Beaulieu, 2018; Zhou et al., 2018). A recent population study utilizing functional MRI data found that children (9–10 years old) exposed to heavy alcohol doses (around 90 drinks consumed during pregnancy) had lower volume and surface area in parietal and temporal lobes compared with children exposed to lighter alcohol doses (around 40 drinks consumed during pregnancy). Strikingly, even the light prenatal alcohol exposure still induced abnormalities in size of brain areas and psychological and behavioral problems (Lees et al., 2020).

Timing of consumption or exposure during pregnancy also plays a role in the symptomology seen in offspring. Alcohol exposure during the first trimester in human pregnancy (gestational day GD 1–10 in rodents) affects the gastrulation and neurulation stages and is associated with characteristic craniofacial dismorphology of FAS, specifically a wider interorbital distance, shorter midface and cranium width. In addition, alterations in cortical volume and alterations in white matter tracts have been seen (Sulik and Johnston, 1983; Lipinski et al., 2012; Parnell et al., 2013; Cao et al., 2014; Petrelli et al., 2018). PAE during the second trimester (GD 11–21 in rodents) leads to midfacial dysmorphologies, smaller skull volume and circumference, and reduction in frontal, parietal and occipital areas (Anthony et al., 2010; Coleman et al., 2012; Kajimoto et al., 2013; Shen et al., 2013). In addition, significant reduction in olfactory bulb and hippocampus volumes has been reported in rodent model (Akers et al., 2011; Petrelli et al., 2018).

The effects observed after alcohol exposure during the third trimester (postnatal day PND 1–10 in rodents) include impairments of the developing visual system, reduction in total brain volume and in total neurons and interneurons number (Dursun et al., 2011, 2013; Coleman et al., 2012). Additionally, neurodevelopmental disorders including cognitive impairments in learning and memory have been reported following third-trimester equivalent exposure (Wilson et al., 2016).

In summary, PAE during gestation alters many crucial and important developmental processes, including neurogenesis, neuronal differentiation and migration (Miller, 1992; Goodlett and Horn, 2001; Guerri et al., 2001, 2009; Olney et al., 2002; Guizzetti et al., 2014). While there is currently a consensus that no amount of alcohol during pregnancy is “safe,” the mechanisms of these alterations seen across development are still not fully understood.

### Cell-Adhesion Molecules: A Possible Target of Alcohol During Development

The Central Nervous System (CNS) is a complex network of interconnected neurons whose efficient structure and functionality requires the establishment of cell-cell interactions both for efficient migration and synaptogenesis (Goodlett and Horn, 2001). These cell-cell interactions are a selective process that requires the presence and function of a class of cell adhesion molecules (CAMs). CAMs may be a critical target of alcohol during development considering the crucial role played in the brain development and in the formation of functional synaptic connections (Washbourne et al., 2004; Li et al., 2017). Astrocytes are now recognized as an essential component of synapses and their interactions with neurons have been shown to be mediated by CAMs. Evidence also suggest that alcohol exposure during development affects neuron-glia interactions (Guerri and Renau Piqueras, 1997; Ullian et al., 2001; Fields and Stevens-Graham, 2002; Yang et al., 2003; Guizzetti et al., 2008; Wilhelm and Guizzetti, 2016; Tan and Eroglu, 2021).

In this review we provide an overview of structures and roles of different types of CAMs involved in CNS development. In addition, we will discuss experimental evidence that PAE impacts CAMs and how these affects may be involved in the sensory, morphological and behavioral features of FASD previously described. The principal aim of the review is to provide a better understanding of how alcohol affects CAMs following PAE, and to identify gaps in the existing literature related to these molecular targets that will further our understanding of how alcohol alters the developing brain.

## Animal and *in vitro* Experimental Models Used for Fetal Alcohol Spectrum Disorders

Studies in animal models of PAE have been an essential tool as the characterization of molecular mechanisms and behavioral alterations associated with FASD are difficult to carry out given the complexity in controlling and measuring variables such as maternal health and nutrition, volume and timing of alcohol exposure during the pregnancy. In order to avoid those limitations, numerous animal and *in vitro* models have been developed.

Rodents are the most widely models used in this field and have demonstrated a high utility in characterizing the PAE effects in the brain and in the complex behaviors. However, the long-lasting developmental, neurobiological and neurobehavioral changes in offspring induced by PAE vary largely depending on the specifics of the model studied (Jones and Smith, 1973; Jones, 1975; Sokol et al., 2003; Chudley et al., 2005; Bird et al., 2015; Marquardt et al., 2020; Licheri et al., 2021). Rodent models have a short gestational period and a large number of offspring (Almeida et al., 2020). In addition, they are useful for investigating the molecular mechanism altered by PAE in relation to exposure time, pattern of exposure and dosage (Almeida et al., 2020). Despite data showing the several similarities in brain architectures and functions between rodent and human brains, rodent models all have limitations. Rodent pregnancy is shorter compared to human pregnancy, and the third trimester equivalent to human gestational period occurs postnatally. Considering the overall size differences across species, in the translational studies it is critical to consider and acknowledge differences in the processes of alcohol administration, distribution, metabolism and elimination.

Several *in vitro* studies have been useful for identifying the molecular mechanisms affected by prenatal alcohol exposure (Lussier et al., 2017). Most of the studies were performed using neural progenitor cells and *ex vivo* primary cell culture (Balaraman et al., 2012; Veazey et al., 2013; Tunc-Ozcan et al., 2016). The *in vitro* models show numerous advantages including the low cost, large number of experimental groups, controlled environments. At the same time with this model is impossible to perform study on alcohol metabolism and on tissue. Recently human cerebral organoids have been developed using induced pluripotent cells grown in Matrigel, a scaffold resembling the extracellular matrix (Lancaster et al., 2013). A newly developed approach, cerebral organoids, which are similar to fetal brains in the aspects of development and structure have also been utilized to examine the role of alcohol on development. For example, recent studies were performed using this model for studying the alcohol effects, in particular the neural pathology phenotypes and signaling pathways (Arzua et al., 2020), including alterations in CAMs (Zhu et al., 2017).

A more recently adopted model of PAE on neural development is the zebrafish (Danio rerio). Although in this model embryonic development is outside of the body, and the probability of alcohol penetration can vary in relation to the concentrations administered (Ali et al., 2011; Meyers, 2018), the short development period and a large amount of offspring have made the zebrafish an important tool in the study of the effects of alcohol on development (Facciol and Gerlai, 2020). Importantly, it has shown strong utility and has yielded important results regarding the effects of PAE on CAMs.

## Role of Cell-Adhesion Molecules in Central Nervous System Development

**FIGURE 1:**
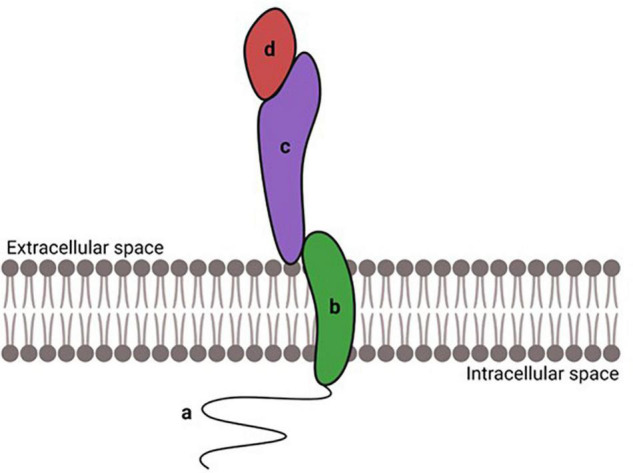
Schematic structure of CAMs. CAMs have an intracellular domain **(a)**, a transmembrane domain **(b)**, and an extracellular domain **(c)** responsible for the binding to ligands **(d)**. Created with BioRender.com.

During the last 50 years various families of CAMs have been described (Rutishauser et al., 1976) and their role in development of brain structures explored (Franco and Müller, 2013; de Agustín-Durán et al., 2021). CAMs play a pivotal role in neural migration, axon growth, and synaptogenesis (Hirano and Takeichi, 2012; Hippenmeyer, 2013; Mandai et al., 2015; Martinez-Garay, 2020). Typically, CAMs are transmembrane proteins with an extracellular domain mediating the interaction with the extracellular matrix (ECM) or other CAMs. The intracellular domain is responsible for the binding to cytoskeleton proteins and for interaction with signal transduction (Chotia and Jones, 1997; Leshchyns’ka and Sytnyk, 2016; Figure 1). Some CAMs are anchored to the plasma membrane through a glycosylphosphatidylinositol (GPI) anchor, called GPI-anchored CAMs. Despite GPI-CAMs do not show the typical intracellular domain, they interact with signal transducer and synapse regulators (Tan et al., 2017). Furthermore, CAMs are classified by whether they are calcium-dependent or calcium-independent; most CAMs are traditionally divided into four groups: the immunoglobulin superfamily (IgSF), the cadherins, the integrins and the selectins (Shapiro et al., 2007). We will briefly review the structure and function of three major classes of CAMs, Neural Cell adhesion Molecules, Cadherins and Integrins, and then discuss evidence that these classes of CAMs are a target of alcohol during development.

## Neural Cell Adhesion Molecule: Structure and Function

Neural cell adhesion molecule (NCAM) is a membrane-bound cell recognition molecule belonging to immunoglobulin (Ig) superfamily (Sytnyk et al., 2017). Its cytoplasmic domain is composed of 110 amino acids, while the extracellular region has five Ig-like and two fibronectin 3 (FN3) domains (Table 1; Grumet, 1997; Kiselyov et al., 2005; Shapiro et al., 2007; Homrich et al., 2015). The Ig-like domains are responsible for homophilic binding (Shapiro et al., 2007), while Ig1 and Ig2 only or all five domains are involved in *trans-*homophilic binding. The NCAM *cis-*homophilic binding is mediated by Ig1 and Ig2, and Ig1 and Ig3 domains (Frei et al., 1992; Ranheim et al., 1996; Atkins et al., 2004). The homophilic interaction is involved in the cell adhesion between the same molecules on membranes of adjacent cells; furthermore, NCAMs can also interact heterophilically with other cell adhesion molecules and proteins of the extracellular matrix (Leshchyns’ka and Sytnyk, 2016).

**TABLE 1:** Structure and role of CAMs during central nervous system development.

Family	Structure	Role
Ig-superfamily (NCAMs, L1)	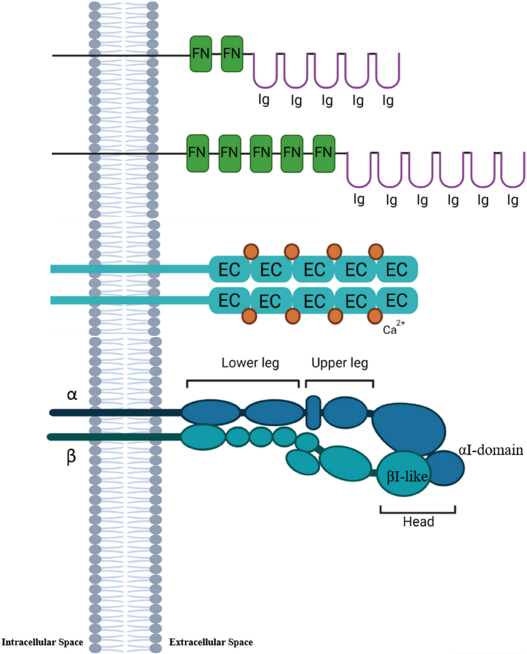	• Cell migration • Cell differentiation • Neurite growth • Axon fasciculation • Synaptic plasticity
	
Cadherins (N-cadherins, E-cadherins)		• Structural integrity of neural tube and cortical structure • Cell migration and synapse formation
	
Integrins		• Cell adhesion • Cell migration • Synaptogenesis • Synaptic plasticity
	

*NCAM, neural cell adhesion molecule; N-cadherin, neural cadherin; E-cadherin, epithelial cadherin. FN, fibronectin domain; Ig, immunoglobulin domain; EC, extracellular cadherin repeat. Created with BioRender.com.*

Three main isoforms have been identified; two are transmembrane forms NCAM-140 and NCAM-180, while the third, NCAM-120 is anchored to the cell membrane through glycosylphosphatidylinositol (GPI) linkage (Cox et al., 2009). Additionally, there is the polysialylated form of the neural cell adhesion molecule called PSA-NCAM; it is well documented that during CNS development, the PSA-NCAM is involved in precursor migration and in neuronal differentiation (Hu et al., 1996; Petridis et al., 2004; Bonfanti, 2006; Rutishauser, 2008; Schuster et al., 2020), and also in the regulation of synaptic plasticity in adult brain (Seki and Arai, 1993; Muller et al., 2000; Saini et al., 2020).

NCAM-140 and NCAM-180 are highly expressed during fetal and postnatal development, respectively (Chuong and Edelman, 1984; Gennarini et al., 1986; Oltmann-Norden et al., 2008), in fact NCAM-140 is localized to growth cones and axon of developing neurons (Sytnyk et al., 2006; Cox et al., 2009). While, NCAM-180 can be found in the postsynaptic membrane of mature neurons (Sytnyk et al., 2006). NCAM-120 is the predominant CAM expressed in glia (Noble et al., 1985), and shows high levels during adult life (Gennarini et al., 1986; Brennaman and Maness, 2008). NCAMs are involved in cell migration and neurite outgrowth through FN3 domains (Schachner, 1997; Crossin and Krushel, 2000; Rønn et al., 2000; Sytnyk et al., 2017; Huang et al., 2020). It has also been shown that NCAMs regulate the synaptic development and plasticity (Muller et al., 2000; Stoenica et al., 2006; Sytnyk et al., 2017; Cameron and McAllister, 2018; Duncan et al., 2021).

Another CAM belonging to immunoglobulin (Ig) superfamily is L1. Similar to NCAM, L1 has an extracellular region composed of six Ig ad five FN3 domains, followed by a transmembrane and an intracellular region containing a specific motif mediating the binding to cytoskeleton (Table 1; Bennett and Baines, 2001; Bian, 2012). The Ig-1 domain mediates the homophilic interactions (Jacob et al., 2002). L1 can interact with other Ig superfamily members through homo- or heterophilic interactions (Jacob et al., 2002; Maness and Schachner, 2007), and ECM molecules, extracellular signal-regulated kinases (Erk), cytoplasmic and traffic proteins (Maness and Schachner, 2007; Bian, 2012). L1 plays an important role during extrinsic signaling transduction regulating cell migration, differentiation, and axon growth through interaction between ECM molecules (Maness and Schachner, 2007). Moreover, the cell-adhesion interactions mediated by NCAM and L1 are calcium-independent process (Bian, 2012). Additionally, some studies have shown the involvement of NCAM and L1 in the axon-fasciculation during early postnatal period (Fischer et al., 1986; Kamiguchi and Lemmon, 1998; Crossin and Krushel, 2000; Barry et al., 2010; Frei and Stoeckli, 2017).

### Neural Cell Adhesion Molecule and Prenatal Alcohol Exposure

Exposure to alcohol during development has been shown to interfere with cell proliferation (Miller, 1996), migration (Miller, 1993), differentiation (Valles et al., 1996) synaptogenesis (Lancaster et al., 1989), gliogenesis (Lancaster et al., 1982), and apoptosis (Liesi, 1997). Common to most of these intrinsic processes, including gene expression and cell-cell interaction, is the involvement of CAMs. Specifically, experiments using *ex vivo* and in *in vivo* models have demonstrated that alcohol exposure during gestational periods affects NCAMs. Almost 30 years ago, it was reported that alcohol exposure (10 mg/50 microliters/day) to chick embryos at embryonic day 1–3 (E1-3) induced a significant increase of PSA-NCAM expression measured via Western blot in cerebral hemispheres between E8 and E10; while no significant change was observed in cerebellum from E10 to E20 (Kentroti and Vernadakis, 1996). Moreover, in cortical cultures NCAMs were found to have altered neuronal growth patterns after alcohol exposure. Neuroblast-enriched cultures obtained from 3-day-old whole chick embryos 3 h post-plating were treated with 50 mM ethanol from 0 to 4 DIV, and then fixed at 3, 6 or 9 DIV. To characterize the possible effects after alcohol exposure, cells were double-stained for NCAM and neurofilament, and the data collected showed changes in growth patterns of developing neurons and an intense NCAM staining. Interestingly, the altered NCAM expression in cerebral hemispheres corresponds temporally with the shift in neuronal phenotype from cholinergic to catecholaminergic and GABAergic (Kentroti et al., 1995; Kentroti and Vernadakis, 1996). Taken together, these data suggest the effects of alcohol on neuronal growth patterns and on NCAM expression might influence the establishment of neurotransmitter phenotype.

The alterations of NCAM expression have been confirmed by a study in rat offspring from dams exposed to alcohol liquid diet [5% (wt/vol) alcohol resulting in 14.3 ± 0.8 g of ethanol/kg/day] before mating, during gestation and lactation. The NCAM expression was measured at different postnatal days using Western blot and it was found that from postnatal day 5 to 7 (PND5-7) the PSA-NCAM showed higher levels in cerebral cortex in alcohol-exposed litters. It was also found that alcohol groups had a delay in the decrease of protein expression between PND7-30 while in parallel, the levels of NCAM-140 and NCAM-180 were significantly reduced (Miñana et al., 2000). In addition, alterations in NCAM isoforms have been observed in primary cultures of cortical neurons obtained from 16-day-old rat fetuses and treated with alcohol (400 mg/dl) for 48 h. This study also found that alcohol exposure significantly up-regulated the expression of NCAM-120 and 140, but not significant modulation of NCAM 180 (Miller and Luo, 2002).

These reported alterations in NCAM expression may change cell-cell interactions that affect neural migration, glial development and synaptogenesis. Even given differences in model, dose and timing of exposure, the findings have shown a considerable and consistent modulation of NCAM after PAE in the cortex. Given the role of multiple cortical subregions in mediating cognitive function, these alterations might mediate impairments in learning and behavioral flexibility induced by gestational alcohol exposure. To our knowledge, there are no studies directly examining the involvement of NCAM in the cognitive deficits induced by PAE in rodent models. However, there is evidence that negative modulation of NCAM function can induce learning and memory deficits (Doyle et al., 1992; Cremer et al., 1994; Sandi et al., 1995). In contrast to these cortical effects, PAE models using a alcohol liquid diet (5%(w/v) ethanol) between gestational days 10 and 21 was shown to alter the distributions of mossy fibers in dorsal hippocampus from rat offspring; but these effects were not mediated by NCAMs (Sakata-Haga et al., 2003).

### Effects of Prenatal Alcohol Exposure on Specific Neural Cell Adhesion Molecule Subtypes

In addition to more global effects of PAE on NCAMs and brain development, several studies have examined the effects of alcohol on specific NCAMs. In order to characterize the effects of alcohol on both L1 and NCAM-140, mouse fibroblasts were transfected with human L1, and treated with 0.3–50 mM of alcohol for 30 min. The aggregation assay reported a significant inhibition of cell-cell adhesion mediated by L1 at 1 mM; in addition, the half-maximal inhibition was observed at 7 mM concentration, while the concentration of 50 mM completely blocked cell-cell adhesion (Ramanathan et al., 1996). Considering that 7 mM is the concentration reached in the blood after 1 or 2 drinks, these data underlying the harmful effects of maternal alcohol exposure induced by light drinking. In the same study, the authors investigated if the alcohol was able to affect the cell-cell adhesion processes also in cerebellar granule cells obtained from PND8 rats, and clinically concentrations of alcohol (5, 10, 25, and 50 mM) were added for 90 min. Here, as well, the authors observed that alcohol inhibited the L1 cell-adhesion, while had no effect on NCAM-140 (Ramanathan et al., 1996). Intriguingly, the same experiments performed in mouse fibroblasts transfected with human NCAM reported that the alcohol exposure did not affect the cell-cell adhesion mediated by NCAM (Ramanathan et al., 1996).

Similarly, Bearer and colleagues demonstrated the inhibitory effect of alcohol at concentration 3–5 mM on L1-mediated neurite outgrowth of cerebellar granule cells obtained from PND6 rats within 12 h (Bearer et al., 1999). PAE effects on L1 in cerebellum has also been investigated *in vivo*. In one study, rat pups on PND6 received 4.5, 5.25 and 6 g/kg of alcohol divided into 2 doses 2 h apart, then sacrificed. Analysis found that the percent of L1 in lipid rafts was significantly increase after alcohol exposure (6 g/kg/day) (Littner et al., 2013). The inhibitory effect mediated by alcohol on L1 adhesion, and the resultant disruption of L1 cell-cell adhesion could justify the accumulation of L1 in the lipid rafts (Tang et al., 2011).

The molecular mechanism behind this inhibitory effect mediated by alcohol on L1-adhesion has also been examined. Cerebellar granule neurons and dorsal root ganglion neurons were exposed to 100 mM of alcohol. Utilizing confocal microscopy the authors reported that alcohol exposure did not affect L1 distribution to the growth cone, while an immunoblot study revealed that the effect of alcohol on L1 is on its activation of pp60^src^ (Yeaney et al., 2009). Interestingly, a similar study performed using cerebellar slices from PND7 rats reported that the treatment with 20 or 100 mM of alcohol for 4, 24 h and 10 days did not alter L1 expression (Fitzgerald et al., 2011).

Importantly, the alcohol-binding site in the extracellular domain of L1 has been identified utilizing photolabeling. This work found that alcohol interacts with this site localized at the interface between Ig1 and Ig4 domains (Arevalo et al., 2008; Dou et al., 2011). Furthermore, the alcohol-inhibition of L1 adhesion can be abolished by decreasing the phosphorylation of serine 1248 (S1248), an Erk2 substrate located to the L1 cytoplasmic domain (Dou et al., 2013). Recently, a study identified three highly conserved sites on L1 cytoplasmic domain involved in L1 sensitivity to alcohol; in fact, Dou and colleagues found that the phosphorylation of L1 cytoplasmic domain at S1152, S1176, S1181, and S1248 promotes L1 coupling with ankyrin-G and spectrin-actin cytoskeleton facilitating L1 sensitivity to alcohol (Dou et al., 2018). In order to determine if there is a correlation between this molecular pathway and susceptibility to FASD, the authors studied the genes involved in phosphorylation of L1 cytoplasmic domain. They found that polymorphisms in genes encoding ankyrin-G and p90rsk, a kinase that phosphorylates S1152 are associated with facial anomalies observed in children exposed to heavy maternal alcohol consumption (Dou et al., 2018).

Taken together, these studies clearly demonstrate that alcohol exposure in particular during the third trimester of gestation inhibits the cell adhesion process mediated by L1 without changes in the protein expression, but through L1-ankyrin G association. Intriguingly, the inhibitory effect is brain region-specific given that the L1 association with lipid rafts is only observable in cerebellum between PND 8–28 (Nakai and Kamiguchi, 2002).

The relation between L1 and FASD is further supported by studies reporting that neuroprotective peptides are able to block alcohol-inhibition of L1 adhesion in C57BL6J mouse embryos. Embryos at gestational day 8 were exposed to alcohol (100 mM), or in combination with the peptides NAPVSIPQ (NAP) and SALLRSIPA (SAL). The incubation with alcohol for 20 h induced neural tube defects, while co-incubation with neuropeptides rescued these alterations (Chen et al., 2005). A more recent study performed combining immunoprecipitation, Western blotting and immunofluorescence in fibroblasts transfected with human L1 demonstrated the protective effect of NAP. In fact, NAP is able to stimulate the phosphorylation of the tyrosine-1229 at the ankyrin binding motif of the L1 cytoplasmic domain, blocking L1-ankyrin-G and spectrin-actin cytoskeleton association through the activation of EphB2, a kinase that phosphorylates L1-Y1229 (Dou et al., 2020). It is already established that the interaction of EphB2 and L1 plays an important role during brain development contributing to the signaling during hippocampal development (Robichaux et al., 2014).

Overall, studies performed in the last 20 years have characterized not only the inhibitory effect of alcohol on L1 adhesion process, but also the molecular mechanism involved and the possible rescue processes. These findings might be useful for future pharmacological approaches. Furthermore, the study performed by Dou and colleagues, where they identified the association between polymorphisms in genes encoding ankyrin-G and FASD facial anomalies, suggests new studies in order to understand if genetic regulation can alter FASD susceptibility (Dou et al., 2018).

As previously mentioned, zebrafish have more recently become utilized as a model of PAE. A study examining the immersion of zebrafish embryos into 1% alcohol solution (vol/vol%) at 24 h post-fertilization for 2 h found that this approach reduced the NCAM expression in different brain regions (Mahabir et al., 2018; Table 2). Interestingly, these data could explain the lower serotonin and dopamine levels observed in zebrafish embryos after alcohol exposure (Buskea and Gerlaia, 2011), considering previous findings reporting the reduction of serotonin transporter protein levels in different brain regions of adult NCAM(-) (/) mice (Aonurm-Helm et al., 2015). Moreover, it has been shown the involvement of NCAM in the trafficking of the neurotransmitter receptor dopamine 2 (Xiao et al., 2009). Taken together these findings demonstrated that different prenatal alcohol exposure models affect significantly the NCAMs in brain region-dependent manner, confirming the role of this class of CAM as like alcohol target during CNS development.

**TABLE 2:** Prenatal alcohol exposure modulates CAMs expression in *ex vivo* and *in vivo* experimental models.

CAM	Experimental model	Doses/treatment	Time of exposure	Effect
PSA-NCAM	Chick embryos	10 mg/50μl/day	E1-5	Increase of protein expression
PSA-NCAM	Rats	5% wt/vol	Before mating, during gestation and lactation	Increase of protein expression
NCAM-140, NCAM-180	Rats	5% wt/vol	Before mating, during gestation and lactation	Reduction of protein expression
NCAM-140, NCAM-180	Cultures of cortical neurons (16 day old fetuses)	400 mg/dl	48 h	Reduction of protein expression
L1	Cultures of cerebellar granule cells (PND8 rats)	5, 10, 25, 50 mM	90 min	Inhibition L1 cell-adhesion
L1	Cultures of cerebellar granule cells (PND6 rats)	3–5 mM	12 h	Inhibition L1- mediated neurite outgrowth
L1	Rats	6 g/kg/day	PND6	Increase in lipid rafts
NCAMs	Zebrafish	1% (vol/vol)	1 day after post-fertilization for 2 h	Reduction of protein expression
Cadherin-8	Mice	25% vol/vol	Gestation	Increase of protein expression
N-cadherin	Chick embryos	2% vol/vol	3 days	Increase of protein expression
N-cadherin	Mice	10% (w/vol)	17 days previous and up to day 10 of gestation	Increase of protein expression
α-integrins and β-integrin 3	Neural progenitor cells	1, 10, 100 mM	3 days	Increase of protein expression
β-integrin 2	Neural progenitor cells	1, 10, 100 mM	3 days	Decrease of protein expression

## Cadherins

The cadherins represent a large family of proteins expressed in simple and complex organisms, many of which participate in Ca^2+^-dependent cell-cell adhesion process. There are more than 100 family members divided in four subgroups including classical cadherins, protocadherins, desmosomal and unconventional cadherins, which have a similar extracellular Ca^2+^-binding region known as extracellular cadherin repeats (ECs) (Table 1; Nollet et al., 2000; Angst et al., 2001; Shapiro et al., 2007; Bian, 2012). Structurally, the classical cadherins are single-pass transmembrane proteins, with a cytoplasmic actin-binding site, while the extracellular site is composed of five EC domains (EC1-5) (Shibata-Seki et al., 2020; Table 1). Cadherins mediate homophilic or heterophilic interaction through a dimer of EC1-5 (Patel et al., 2006; Brasch et al., 2018). Classical cadherins are further divided into type I and II based on sequence comparison. The first group includes neural cadherins (N-cadherins) and epithelial cadherins (E-cadherins), which have a conserved histidine-alanine-valine (HAV) amino acid sequence in the distal EC (EC1), which is important for homophilic adhesion (Takeichi, 1995; Halbleib and Nelson, 2006). Moreover, type I class mediates a strong cell adhesion (Paulson et al., 2014). In contrast, type II classical cadherins do not have a HAV motif, and consequently are associated with less strong cell-adhesion (Thiery et al., 2012). Furthermore, the catenins connect N-cadherin to the actin cytoskeleton mediating the cadherin-mediated cell-adhesion (Meng and Takeichi, 2009; Takeichi, 2014).

N-cadherin plays an important role in maintaining the structural integrity of the neural tube and cortical structure during development (Radice et al., 1997; Kadowaki et al., 2007; Punovuori et al., 2021). The complexity of the CNS depends on key role played by N-cadherin, which controls cell migration, synapse formation and maintenance of progenitor pool (Togashi et al., 2002; Bekirov et al., 2008; Rieger et al., 2009; Camand et al., 2012; de Agustín-Durán et al., 2021). For this reason N-cadherin levels are tightly regulated, and upregulation and downregulation can lead to significant alterations during CNS development. The overexpression of N-cadherin inhibits the differentiation of neural progenitors, while downregulation induces a premature differentiation (Barami et al., 1994: Rousso et al., 2012).

Additionally, E-cadherins are required for cell movement during gastrulation (Babb and Marrs, 2004; Kane et al., 2005; Solnica-Krezel, 2006; Morita and Heisenberg, 2013; Song et al., 2013), and for developmental signaling pathways, including including non-canonical Wnt (Ulrich et al., 2005), heterotrimeric G-protein (Lin et al., 2009) and Pou5f1/Oct4 signaling pathways (Song et al., 2013).

Numerous studies have demonstrated the pivotal role played by Type I and Type II cadherins in the formation of specific synaptic connections, in fact they are localized in both pre- and post-synaptic terminals (Bekirov et al., 2002; Arikkath and Reichardt, 2008; Williams et al., 2011; Basu et al., 2015). A recent study described the important role of cadherins in developing CNS including the organization into layers and the formation of neuronal circuits (Polanco et al., 2021).

### Prenatal Alcohol Exposure Affects Cadherin Expression and Interactions

During CNS development, cadherin 8 is expressed in frontal and motor cortices (Dye et al., 2011), and prenatal alcohol exposure is able to alter the protein level of this CAM. Cadherin 8 is a classical type II cadherin (Kido et al., 1998), and during the perinatal and postnatal period it shows a restricted expression patterns in specific brain regions including cortex, hippocampus and striatum (Medina et al., 2004; Lefkovics et al., 2012). To evaluate the effect of prenatal alcohol exposure on cadherin 8, El Shawa and colleague exposed mouse dams to alcohol (25% v/v) for the entire gestational period (GD 0.5-GD 19.5). Pups were sacrificed at postnatal day 0, and *in situ* RNA hybridization showed a significant increase of cadherin 8 expression in cortex (El Shawa et al., 2013). The upregulation of N-cadherin induced by alcohol exposure was further supported in a study where dams mice were exposed to alcohol 10% solution for 17 days before and up to day 10 of gestation (Coll et al., 2011). Western blotting data showed a significant increase of E-and N-cadherins levels in E10 embryos coming from dams exposed to alcohol (Coll et al., 2011).

Disruptions in cadherins have also been shown in zebrafish models. Zebrafish embryos exposed to 100 mM (0.6% vol/vol) alcohol from to 2 to 8 h post-fertilization (hpf), and it was shown that following 4.5 of exposure alcohol affected yolk cell microtubule and E-cadherin distribution (Sarmah et al., 2013). Alcohol effects on cell adhesion process were still observed at 8 hpf (mid-gastrulation) and microarray analysis showed a reduction in gene expression of protocadherin-18a (Pcdh-18a), suggesting that alcohol exposure continued to affect cell-cell communication in treated embryos (Sarmah et al., 2013).

Approximately 30 years ago, the class of protocadherin was identified and described similar to cadherins, but with the difference that they show six or seven ECs (Sano et al., 1993). Despite this structural difference, similar to cadherins, the protocadherin expression is regulated during CNS development, and they play an important role during the specification of neuronal identity (Pancho et al., 2020). Recently, a study performed using chick embryos as experimental model reported that alcohol exposure (2% alcohol) once every 1.5 days for 11 days upregulated the N-cadherin and cadherin 6B expression, and downregulated cadherin 7 in dorsal neural tube (Zhang et al., 2017). These alterations in the protein expression may inhibit neural crest cells migration leading to the craniofacial defects (Zhang et al., 2017; Table 2).

Despite the different experimental models used, the data collected clearly demonstrated that alcohol exposure modulates cadherin expression during early development. Overall, the data suggest that abnormal regulation of cadherin expression could explain the abnormalities observed in the neural tube following developmental alcohol exposure.

## Integrins

Integrins are a class of CAMs that are major mediators of cell-cell adhesion and cell-ECM interactions (Barczyk et al., 2010; Ringer et al., 2017). Integrins are heterodimeric transmembrane proteins consisting of α and β subunits, and electron microscopy studies show that this class of CAMs has a globular head and two leg regions (one formed by α subunits and the other by β subunits; each region is subdivided in lower and upper leg) into the plasma membrane (Table 1; Srichai and Zent, 2010). Each subunit has an extracellular domain, a transmembrane domain, and a cytoplasmic tail (Srichai and Zent, 2010). The α subunits are responsible for ligand binding, while both α and β subunits mediate the cell signal transduction (Srichai and Zent, 2010; Pan et al., 2016). The integrin α subunits can be further divided according to the presence of an I-domain, a crucial motif for ligand binding. The extracellular domains of integrin α subunits have a “metal-ion-dependent adhesive site” (MIDAS) that is able to bind divalent metal cations, while the transmembrane domains through 5 common amino acid sequences “GFFKR” regulate integrin affinity by mediating an alpha-beta subunit cytoplasmic tail interaction (Pan et al., 2016).

The structure of integrin β subunits is well described as an I-like domain similar to I-domain characterized in α subunits. This I-domain is a highly conserved region composed of 240 residues, and it contains two additional sections that either are responsible for ligand binding (Huang et al., 2000; Xiong et al., 2002). Moreover, they show a large extracellular domain, a single-spanning transmembrane domain and a cytoplasmic tail (Pan et al., 2016).

Importantly, integrins are expressed in brain areas heavily involved in learning, memory and cognition including the hippocampus, cerebellum, thalamus and cortex (Pinkstaff et al., 1999; Clegg et al., 2003). There is strong evidence that these proteins mediate the adhesion and migration of neuronal cells during the developing of CNS (Galileo et al., 1992; Zhang and Galileo, 1998; Anton et al., 1999; Clegg et al., 2003). Interestingly, it has been reported that the neuronal migration during CNS development is modulated also by the interaction with integrins and the L1 (Silletti et al., 2000; Thelen et al., 2002).

Several integrin α and β subunits are highly expressed in growth cones and synapses (Wu and Reddy, 2012; Park and Goda, 2016), and it is well established their role in building and maintain synaptic structure during the phases of the development (Benson et al., 2000). *In vitro* studies have also shown that the integrins are able to stabilize long term potentiation (LTP) after induction (Stäubli et al., 1998; Babayan et al., 2012; Kerrisk et al., 2014), therefore, integrins may be another CAM target of alcohol considering their role during brain development.

### Integrins and Prenatal Alcohol Exposure Effects

Similarly to its CAM family members, there is some evidence that integrins are affected by alcohol exposure during development. Pharmacological concentrations of alcohol (1, 10, and 100 mM) added to cultures of neural progenitor cells for 3 days modulate the mRNA levels of genes involved in the cell adhesion pathways. In particular, the expression of α integrin 5 and β integrin 3 was significantly increased, while the β integrin 2 was downregulated (Vangipuram et al., 2008). Considering that integrins play a pivotal role in cell proliferation and migration during brain development (Schmid and Anton, 2003), another experimental investigation focused its attention on the integrin protein levels in fetal cortices. The dams were exposed to alcohol (from 6 to 17 gestational days) using a liquid diet with increasing concentrations of alcohol (v/v), precisely during GD 6 and 7 the concentration was 2.2%, then increased to 4.5% during GD 8, 9 and 10, and 6.7% during GD 11 to 19. The offspring cortices analyzed at gestational day 18 showed a significant increase of β integrin 1, while a reduction for α integrin 3 (Rout and Dhossche, 2010; Table 2). These results could explain PAE’s effects on thickness of cortical areas measured in mouse offspring (Abbott et al., 2016), confirming the involvement of integrins in the formation of cell layers in cortex.

While there is some evidence that integrins may be altered by PAE, less work has focused on this class of CAMs than either the NCAMs or cadherins. Considering the important role by this class of cell adhesion molecules plays not only during CNS development but also in learning and memory processes, further investigation is needed in order to evaluate the possible involvement of integrins in the cognitive deficits observed in FASDs.

## Areas of Future Focus

In the last 50 years, the impact of alcohol consumption during pregnancy has been extensively investigated, but the molecular mechanisms underlining abnormalities observed in PAE offspring are still not understood. Given the evidence reviewed, it can be well established that alcohol also effects on cell-cell interaction, in particular on cell adhesion molecules. However, several areas are identified that need more focus to understand how PAE affects these molecules.

### Effects of Prenatal Alcohol Exposure Variables

Despite CAMs being expressed throughout the brain, the literature to date suggests that PAE modulates the protein level expression in a region-specific manner. NCAMs expressed in cortex are most consistently affected by alcohol exposure, although the dosage, duration and gestational timing of PAE models used all impact these effects. Since few studies have been performed so far, future investigation will be needed to evaluate the PAE’s effects in rodent model using different doses, time of exposure and routes of administration. To date, the literature specific to dose and model suggests that both low, moderate and high alcohol exposure paradigms in rodent models lead to significant alterations in the protein expression of different CAMs, in particular NCAMs and N-cadherins. Given that recent studies have identified gestational day (G12) as a vulnerable stage during fetal development, especially for anxiety-like behavior in offspring (Rouzer et al., 2017), it would be interesting and useful to evaluate the impact on CAMs after a single day of alcohol exposure in rodent models, considering the limits described in the section about *in vitro* studies. Moreover, it is well established that single P7 alcohol exposure reduces total brain volume in adult animals P80 (Coleman et al., 2012). Considering these interesting experimental findings and the role played by the CAMs during the development, it would be appropriate to evaluate the possible PAE effects in relation to the time exposure.

### Interactions of Sex and Prenatal Alcohol Exposure on Cell Adhesion Molecules

The collected data to date reveal an important gap present in literature concerning the role of sex on PAE effects on NCAMs, cadherins and integrins. At the time of this review none of the studies performed in rodent models discussed here have investigated the possible sex differences in expression of CAMs following PAE. Recent focus on sex specific effects in PAE models have revealed it to be a critical biological variable in several widely utilized exposure models. For example, in a model of third-trimester exposure (two injections of alcohol (20% w/v) 2 h apart on P7) adult hippocampal neurogenesis was shown to be altered in a sex-specific manner (Coleman et al., 2012). Similarly, “drinking in the dark exposure” during first and second trimester equivalent was found to impair visuospatial discrimination robustly in females, but not in males (Kenton et al., 2020). That same exposure model was recently shown to alter evoked *N*-methyl-D-aspartate (NMDA) currents in orbital frontal cortex pyramidal neurons in a sex specific manner (Licheri et al., 2021). Considering the important role played by CAMs in synaptogenesis, it might be possible that this sex-specific effect could be mediated by molecular mechanisms involving this class of molecules. Interestingly, recent studies describe sex-related changes in dendritic and synaptic architecture during human brain development (Duerden et al., 2020). In addition, sex differences were seen in the gene expression levels of postsynaptic cell-adhesion in rats between P5 and P7 days (Srancikova et al., 2021). Together, these studies underline the importance of investigating the role of sex in the effects of alcohol on cell-adhesion molecules in morphological, sensory and cognitive effects in FASD.

## Conclusion

There is strong evidence across several preclinical models, and from limited clinical studies, for the involvement of CAMs in the development of neurobiological abnormalities and behavioral effects following PAE. While more work needs to be done to disentangle the role of specific CAMs in these processes, the potential for this class of proteins for developing pharmacological therapies makes this an important area of research focus going forward.

## Author Contributions

VL performed literature search and outline, and wrote the review. JLB contributed to the final draft. Both authors revised the final draft for important intellectual content.

## Conflict of Interest

The authors declare that the research was conducted in the absence of any commercial or financial relationships that could be construed as a potential conflict of interest.

## Publisher’s Note

All claims expressed in this article are solely those of the authors and do not necessarily represent those of their affiliated organizations, or those of the publisher, the editors and the reviewers. Any product that may be evaluated in this article, or claim that may be made by its manufacturer, is not guaranteed or endorsed by the publisher.
